# Usability of cooperative surgical telemanipulation for bone milling tasks

**DOI:** 10.1007/s11548-020-02296-8

**Published:** 2020-12-23

**Authors:** Philipp Schleer, Manuel Vossel, Lotte Heckmann, Sergey Drobinsky, Lukas Theisgen, Matías de la Fuente, Klaus Radermacher

**Affiliations:** Helmholtz Institute for Biomedical Engineering, RWTH Aachen, Pauwelsstraße 20, 52074 Aachen, Germany

**Keywords:** Surgical robotics, Synergistic systems, Shared control, Robotic manipulators, Human machine interaction, Haptics

## Abstract

**Purpose:**

Cooperative surgical systems enable humans and machines to combine their individual strengths and collaborate to improve the surgical outcome. Cooperative telemanipulated systems offer the widest spectrum of cooperative functionalities, because motion scaling is possible. Haptic guidance can be used to assist surgeons and haptic feedback makes acting forces at the slave side transparent to the operator, however, overlapping and masking of forces needs to be avoided. This study evaluates the usability of a cooperative surgical telemanipulator in a laboratory setting.

**Methods:**

Three experiments were designed and conducted for characteristic surgical task scenarios derived from field studies in orthopedics and neurosurgery to address bone tissue differentiation, guided milling and depth sensitive milling. Interaction modes were designed to ensure that no overlapping or masking of haptic guidance and haptic feedback occurs when allocating information to the haptic channel. Twenty participants were recruited to compare teleoperated modes, direct manual execution and an exemplary automated milling with respect to usability.

**Results:**

Participants were able to differentiate compact and cancellous bone, both directly manually and teleoperatively. Both telemanipulated modes increased effectiveness measured by the mean absolute depth and contour error for guided and depth sensitive millings. Efficiency is decreased if solely a boundary constraint is used in hard material, while a trajectory guidance and manual milling perform similarly. With respect to subjective user satisfaction trajectory guidance is rated best for guided millings followed by boundary constraints and the direct manual interaction. Haptic feedback only improved subjective user satisfaction.

**Conclusion:**

A cooperative surgical telemanipulator can improve effectiveness and efficiency close to an automated execution and enhance user satisfaction compared to direct manual interaction. At the same time, the surgeon remains part of the control loop and is able to adjust the surgical plan according to the intraoperative situation and his/her expertise at any time.

## Introduction

Machines offer an excellent geometric accuracy; however, their abilities in complex and unstructured environments are still limited. Humans on the contrary are able to work in unstructured environments with complex stimuli and process qualitative data [1, 2]. Cooperative or synergistic systems try to exploit these synergies for a dynamic interactive motion control, which makes an adequate human–machine communication even more important. Within the cooperative spectrum, a recent study found that telemanipulative devices, where a so-called slave device is remote controlled by a master manipulator, offer the widest variety of functionalities because motion scaling is possible [3]. Furthermore, haptic guidance can be used to transfer information from the preoperative plan directly to manual motion control, while avoiding common bottlenecks of eye-hand coordination [4–7]. Thereby, effectiveness and efficiency can be improved while reducing the workload for the surgeon [8–12]. A frequently mentioned drawback of commercially available telemanipulation systems is that surgeons are not able to feel the acting forces at the slave, so-called haptic feedback. The latter can be used to differentiate tissues and it is scientifically attributed with an increased precision and reduced error rates and reduced force peaks [13–16]. However, if haptic guidance and haptic feedback are combined on the same haptic interface, overlapping and masking of the forces can occur [17, 18]. Therefore system designers have to pay close attention when allocating information to the haptic channel. For a more comprehensive overview regarding surgical usability the reader is referred to [3].

In orthopedics and neurosurgery, there are several examples where either haptic guidance, haptic feedback or both are important. In this respect three exemplary surgical scenarios are investigated, which are tissue differentiation, guided millings such as unicompartmental knee arthroplasties (UKA) and depth sensitive millings performed during laminectomy or craniectomy. Further details with respect to the applications are described along with the experimental description. The aim of the study is thus the evaluation of different cooperative modes of a developed cooperative surgical telemanipulator in comparison to the direct manual or fully automated execution with respect to usability.

## Materials and methods

Three experiments modelling the abstracted surgical tasks *tissue differentiation*, *guided milling* and *depth sensitive milling* were designed to evaluate the MINARO^HD^ surgical telemanipulator. The telemanipulator setup, the different experiments and the experimental procedure will be further described in the following.

### Telemanipulator setup and manual milling

**Fig. 1:**
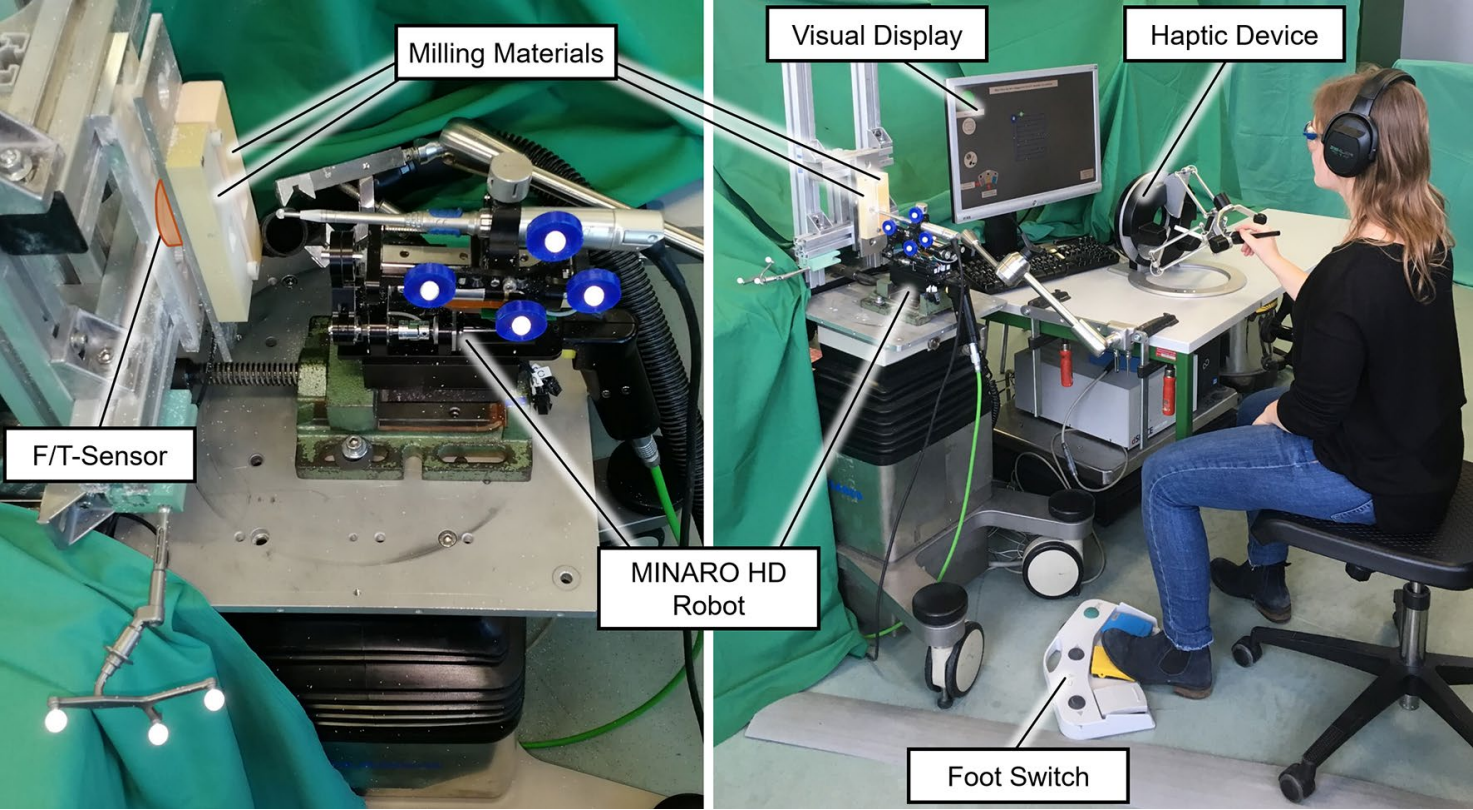
Overview of the telemanipulator setup (right) including the visual display, the haptic device, milling materials, foot switch, the MINARO^HD^ robot and a close-up of the slave side (left) including placement of optical markers

The master device consisted of an omega.6 haptic device (Force Dimension, Nyon, Switzerland) in association with the real-time development software QUARC (Quanser, Markham, Canada). The MINARO^HD^ was used as the slave robot, which is controlled by a DS1006 processor board (dSpace, Paderborn, Germany) [19]. Positions of the Aesculap HiLAN milling tool used with a fixed rotation speed of 60,000 rpm and a 6 mm rosen burr (Aesculap AG, Tuttlingen, Germany) were tracked by a fusionTrack 500 optical tracking system (Atracsys LLC, Puidox, Switzerland). Optical markers were placed on the milling material attachment and the milling tool (compare Fig. 1) to calculate the position of the burr in the milling material coordinate system. Each milling tool was used for 5 participants in order not to influence results by wear of the milling tool. Acting forces were measured by a FTS-Mini-45 force torque sensor (SCHUNK GmbH & Co. KG, Lauffen/Neckar, Germany) and its analogue signals were read by a DAQ card (NI PCI 6221, National Instruments, Austin, USA). Control loops of master (i.e. QUARC) and slave (i.e. dSpace processor board) both ran at an update frequency of 1 kHz and information were exchanged by an RS-422 connection. Additionally, a foot switch (steute Technologies GmbH & Co. KG, Löhne, Germany) and a computer keyboard are used for user input commands. Two materials, obomodulan® 1200 sahara (OBO-Werke GMbH, Stadthagen, Germany) and SikaBlock® M330 (Sika Deutschland GmbH, Bad Urach, Germany) were chosen based on their densities to simulate a hard and a soft material similar to compact and cancellous bone, respectively, (based on the standard for rigid polyurethane foam materials for testing of orthopedic devices (ASTM F1839-08 2016) [20] and the density of human bone [21–24]). The whole setup is depicted in Fig. 1.

For manual milling participants held the Aesculap HiLAN milling tool with a fixed rotation speed of 60,000 rpm, which was equipped with an optical marker, directly in their hand and performed the tasks described in the following.

### Tissue differentiation

The first experiment investigates whether subjects are able to differentiate between compact and cancellous bone based on the haptic feedback provided. Therefore, participants milled into both materials mounted next to each other, both manually and using the telemanipulation system, and were asked to indicate the harder one. For the manual milling materials were mounted horizontally on a measuring table. Subjects were free where and how much they milled during the experiment. Hearing protection was used and a visual barrier was placed between the participants and the milling materials to limit auditory and visual influences on the decision. Afterwards, participants were queried on the difficulty to distinguish materials and if perceptions differed directly manually and telemanipulatively on a seven-level Likert scale (1≙disagree and 7≙agree). The sequence in which participants performed the first experiment (i.e. manually or teleoperated) as well as the side on which a material was presented was randomized.

Haptic feedback during telemanipulation was provided in the depth direction only. The other two degrees of freedom (DOF) of translation are expected to be occupied by haptic guidance in a milling scenario (see third experiment on depth sensitive milling) as the allocation of haptic guidance and haptic feedback to separate DOF is a possibility to avoid superposition of forces [11]. For haptic feedback control a direct force reflection (DFR) controller was used for the soft material, which is a widely used control architecture where position commands are sent from master to slave and forces measured between slave and environment are sent back to the master [25]. DFR is attributed with good tracking as long as the time delay is low, a correct stiffness perception as well as a negligible position drift [26]. However, stability problems are encountered in hard contacts [25]. Therefore, stiffness reflection (SR) control is chosen for the hard material [27]. However, SR control only considers forces in the direction of movement but due to the burr geometry (i.e. rosen burr) forces in the depth direction also arise during lateral movement. Therefore, forces due to lateral movement are fed back directly scaled on basis of the direction of the velocity vector. For safety purposes an additional velocity limiter to avoid excessive milling forces or temperature rise which can lead to bone damage was implemented [28].

### Guided milling

The following experiment modelled tasks such as unicompartmental knee arthroplasties (UKA) where a defined volume has to be removed. Thereby, accurate milling of the cavity is important to ensure a good prosthesis fit [29]. Malalignment can lead to excessive wear and loosening of the prostheses components [30]. During UKA intraoperative referencing is sufficient such that the surgeon can be assisted in three degrees of freedom during the whole execution. Similar 3D volume milling tasks can be observed in neurosurgery, craniofacial surgery or ENT (ear, nose and throat) surgery.

Therefore, participants were asked to mill a cuboid of about 20 mm side length and a depth of 3 mm as accurately as possible, while optimizing execution time as well, as a secondary goal. Each participant performed the milling directly manually (M) as well as using two telemanipulated modes, namely *constraint* (C) and *trajectory* (T) mode, both in the soft and the hard material. After each mode subjects filled out the System Usability Scale (SUS) questionnaire containing 10 questions which are answered on a five-level Likert scale to calculate a variable from 0 (low usability) to 100 (high usability) which indicates the perceived usability of the system [31]. Additionally, the NASA-TLX rating scales where participants indicate their mental, physical and temporal demand as well as their performance effort and frustration on a scale from 1 to 20 [32] were filled out by the subjects following each mode. At the end of the guided milling experiment subjects were asked to fill out the NASA-TLX source of workload which is 15 pairwise comparisons to weigh the separate dimensions of the NASA-TLX to calculate the overall task workload from 0 (low workload) to 100 (high workload) [32]. Besides, participants were asked to rate the following statements on a seven-level Likert scale (1≙disagree and 7≙agree):

- I had full control over the execution of the task.
- I paid close attention to the visual display while milling. (not asked for manual execution)
- I paid close attention to the milling robot while milling. (not asked for manual execution)

Additionally, an exemplary automated milling (A) was performed, independent of the user study, as a reference for effectiveness and efficiency.

For the direct manual milling, the materials were positioned horizontally on a measuring table and the outline of the target volume was drawn on the materials. To check the target depth participants got a cuboid with a thickness of 3 mm for visual comparison.

The first teleoperated mode, the *constraint* mode, was chosen based on findings of a previous study [33]. In this mode the user is haptically constrained within a volume such that movements exceeding the boundary are counteracted by artificial guidance forces generated by a unidirectional PD controller with *P* = 5.4 N/mm and *D* = 0.03 Ns/mm in the depth direction and *P* = 2 N/mm and *D* = 0.05 Ns/mm in the main visual plane. Additionally, haptic velocity limits are implemented with respect to recommended feed rates in the literature scaled to master velocities (soft material: depth = 13.2 mm/s, planar = 25 mm/s; hard material: depth = 4.4 mm/s, planar = 16 mm/s compare [28, 34–38]).

**Fig. 2:**
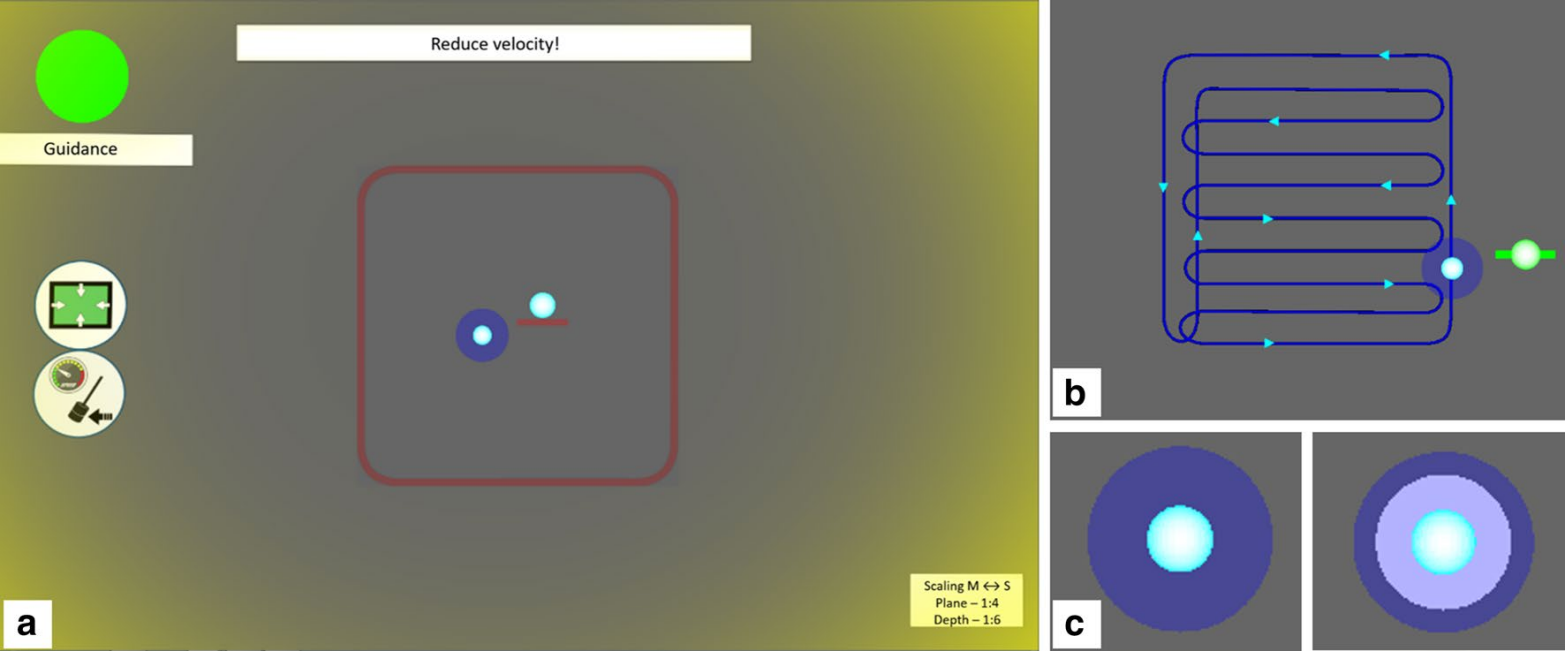
GUI for guided milling experiment **a** main view in constraint mode when velocity is exceeded, **b** trajectory (second layer), **c** visually substituted haptic feedback

The second teleoperated mode was the *trajectory* mode, which was found to increase efficiency as well as perceived system usability, while reducing the perceived workload for the user [33]. In this mode users were guided along a milling path in two layers. In the upper layer the cavity is brought to a depth of 1.5 mm. Once the first layer is completed a vibrotactile signal (amplitude = 0.3 N, frequency = 100 Hz, duration = 333 ms) indicates the transition to the second layer and the user is haptically constrained in space, while the depth is lowered onto the target depth of 3 mm. The individual segments of the path of the second layer (Fig. 2) are closer together to create a smoother surface. Haptic guidance along the trajectory is implemented based on a proxy method to avoid skipping of the reference point. Deviations are counteracted by a PD controller with *P* = 5.4 N/mm on an overshot in the depth direction and *P* = 4.5 N/mm in the opposing direction to ease movement in less critical directions. Movements in the main visual plane are constrained with *P* = 1.5 N/mm. All damping values are set to *D* = 0.03 Ns/mm. Additionally, in case the trajectory is active users can activate a velocity guidance (soft material: *v* = 25 mm/s; hard material: *v* = 16 mm/s) by pressing and holding down the yellow foot pedal. For velocity guidance a PD controller (*P* = 0.2 Ns/mm, *D* = 0.001 Ns^2^/mm) with respect to the target velocity is used with a force output limit of 2 N such that users are always able to counteract the guidance force and an uncomfortably strong pull along the trajectory is avoided. If the velocity guidance is not activated the haptic velocity limit of the constraint mode is active. Pressing the grey foot pedal deactivated the trajectory guidance and replaced it by the constraint mode, because the possibility to switch off the trajectory improves the perceived influence on task execution [33]. By pressing the grey foot pedal again, the trajectory mode could be reactivated.

To make forces acting on the slave side transparent to the operator, the amplitudes of these forces are visually displayed to the operator (Fig. 2) to avoid overlapping and masking of the feedback and guidance forces. Feedback forces in the depth direction were displayed as a white overlay on the cursor which varied in size. Furthermore, boundaries were highlighted when they were violated to support understanding of guidance forces [e.g. the outer contour of the constraint is highlighted if it is exceeded; a yellow frame at the edges of the screen is displayed if the velocity limit is exceeded (Fig. 2)]. Furthermore, a smaller cursor floating next to the main cursor was implemented to visualize the current depth and active assistances or—if applicable—haptic feedback modes were visualized on the side of the screen.

Dependent variables of the experiment were chosen based on the general requirements for basic safety and essential performance specified in part 1–6 of the standard for medical electrical equipment (DIN EN 60,601–1-6) [39] and constituted of:

- *Effectiveness-*mean absolute depth error and mean absolute deviation from contour (main visual plane) evaluated using the data of the optical tracking system. Mean absolute depth error was calculated per participant by averaging the depth error including the diameter of the burr with respect to the planned depth for each data point. The absolute deviation from the contour was calculated per participant by averaging the absolute deviation between the planned contour and the reconstructed contour including the diameter of the burr for each data point.
- *Efficiency-*duration of milling; Participants started and stopped the timer by pressing a button on the keyboard for all modes. Additionally, in the teleoperation modes participants were haptically constrained to a designated starting point before the measurement was started.
- *User satisfaction-*based on NASA-TLX and SUS

### Depth sensitive milling

The last experiment addressed interventions such as laminectomy or craniectomy where the outer contour of the bone to be removed is defined and sensitive tissues such as e.g. the dura mater lie underneath. However, due to inaccuracies of sensor data underlying the planning information (e.g. CT-image resolution typically limited to ± 0.5 mm) the surgeon cannot be assisted underneath a security offset of approximately 1 mm [37]. No assistance is offered in the depth direction within the security offset and the surgeon has to rely on visual and, if offered, haptic cues, which contain important supplementary information due to a distinct force profile during milling [40].

Therefore, a plate of about 4 mm thickness was manufactured out of the hard material and mounted such that there is free space behind. Participants were asked to fully remove a volume of about 28 × 8 mm side length and a depth equaling the thickness of the plate. They were advised not to exceed the lower edge with the burr. Each participant performed the milling manually and using two teleoperated modes. For the manual milling the material was mounted horizontally on a measuring table and the outer contour of the cavity was drawn onto the material. In the teleoperated modes participants were first guided using a trajectory with a haptic velocity limit in two layers to remove the first 3 mm. For the last layer of 1 mm (which corresponds to the security offset mentioned above [37]) the modes consisted either solely of the visual substitution of the force (V) as explained before—or additional haptic feedback in the depth direction was provided (H + V). The outer contour of the cavity was haptically constrained in the main visual plane. These two configurations were chosen since a previous study showed that the visual substitution provides superior effectiveness and additional haptic feedback increases perceived usability [33]. Following each mode participants were asked to fill out a questionnaire containing the SUS questionnaire and the NASA-TLX rating scales. Upon completion of the experiment participants filled out the NASA-TLX source of workload questionnaire.

Dependent variables of the experiment were chosen based on DIN EN 60601–1-6 [39] and constituted of:

- *Effectiveness* mean overshoot evaluated using the data of the optical tracking system. The mean overshoot was calculated per participant by averaging the depth error including the diameter of the burr with respect to the measured thickness of the plate for each data point.
- *Efficiency* duration of milling similarly acquired as during the experiment on guided millings
- *User Satisfaction* based on NASA-TLX and SUS

During all experiments movements of the slave robot in the telemanipulated configurations were scaled down by a factor of 6 in the depth direction and a factor of 4 in the remaining DOFs.

### Procedure

**Fig. 3:**
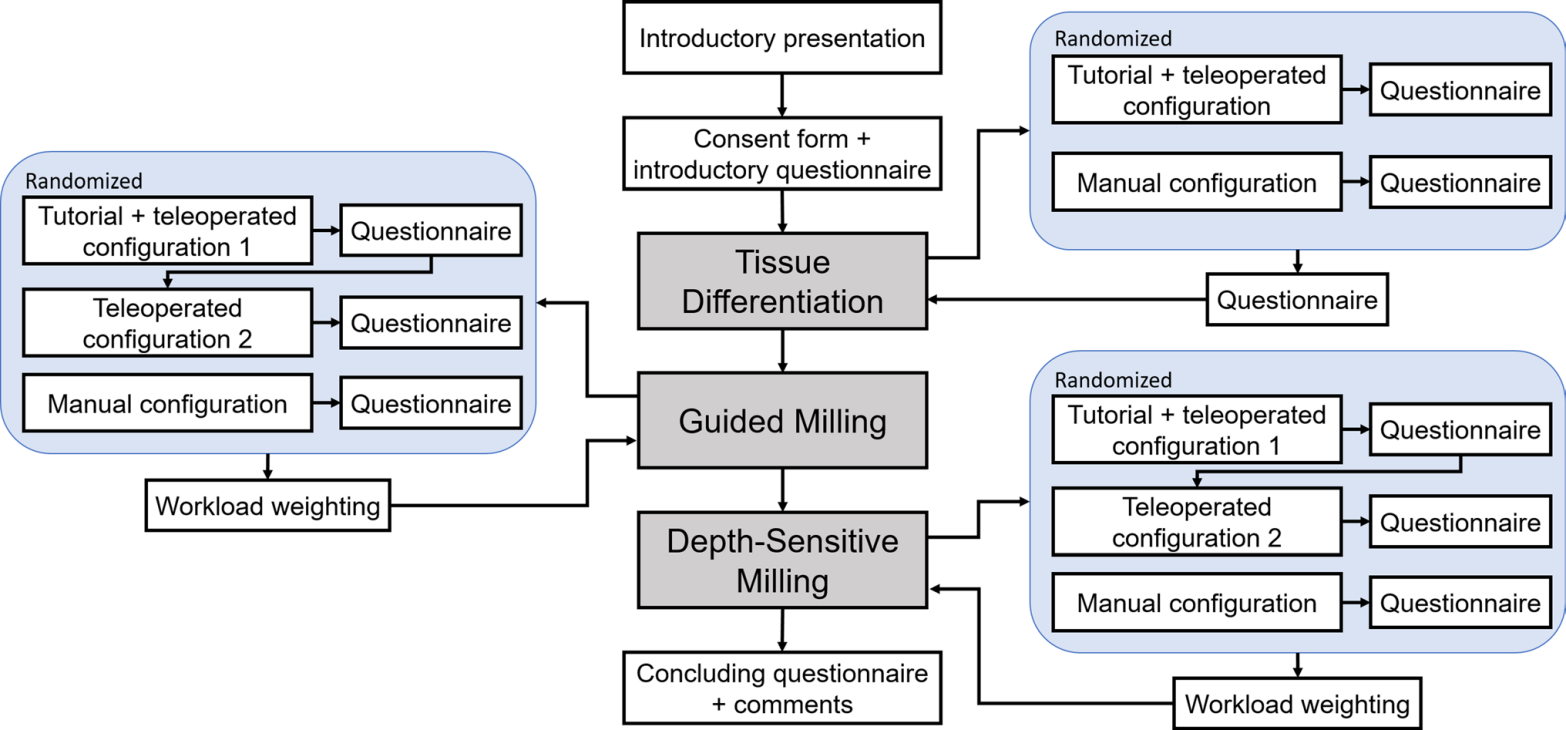
Scheme of the user study

The sequence of the experiments is visualized in Fig. 3. In the beginning, participants were briefed by a short introductory presentation to convey information relevant to the tasks. Informed consent was obtained and participants filled out an introductory questionnaire. Afterwards, the three experiments were conducted sequentially while modes and if applicable the order of materials (i.e. soft or hard) within each experiment were randomized. However, to reduce changeover times, after testing either one of the teleoperated modes the other teleoperation mode was tested. Before the respective first teleoperated mode a short tutorial was presented with the slave robot disengaged to introduce subjects to the user interface. Upon completion of the whole study participants were asked to fill out a final questionnaire with respect to their overall impression including the rating of the following two statements on a seven-level Likert scale (1≙disagree and 7≙agree):

- I could imagine performing manual/teleoperated milling for a prolonged time (15–30 min).
- The manual/teleoperated milling was physically demanding or uncomfortable.

20 non-medical participants took part in the experiments aged between 23 and 55 (5 females, 15 males, 2 left-handed) with an average technical affinity (based on [41]) of 5.13 out of 6. Statistical evaluation was performed using analysis of variance (ANOVA) with a post hoc test using the Tukey–Kramer method with an alpha level of 0.05.

## Results

Results are presented in the following structured with respect to the three performed experiments, tissue differentiation, guided milling and depth sensitive milling.

### Tissue differentiation

In the first experiment all subjects correctly identified the harder material, excluding two who stated that their correct decision was strongly dependent on other factors (i.e. seeing the material despite the visual barrier). Subjects on average agreed that it was easy to distinguish the materials (agreement to ‘The materials were difficult to distinguish’ of 1.8 and 2.9 out of 7 for manual and teleoperated milling, respectively). Furthermore, participants slightly agreed with an average of 4.3 ± 1.9 for both, soft and hard material, that the materials felt similar manually and using the telemanipulation setup.

### Guided milling

**Fig. 4:**
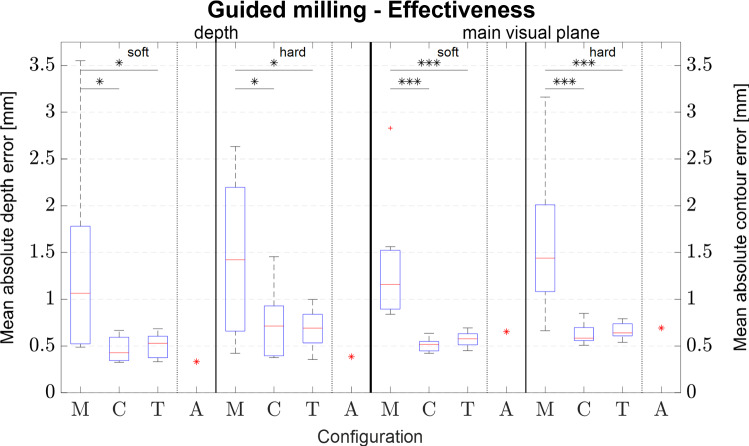
Effectiveness measured by mean absolute error in slave coordinates for the different materials (*p < 0.05, **p < 0.01, ***p < 0.001): M ≙ manual; C ≙ constraint; T ≙ trajectory; A ≙ automated)

Results regarding effectiveness are visualized in Fig. 4. Due to insufficient tracking data (e.g. tracker concealment) several subjects had to be excluded resulting in 7 to 11 data sets per configuration for the effectiveness measure. Statistically significant differences were found in the depth direction for the soft (*F*(2,23) = 5.347; *p* = 0.012) and hard (*F*(2,23) = 5.656; *p* = 0.010) material as well as in the main visual plane for soft (*F*(2,23) = 14.298; *p* < 0.001) and hard (*F*(2,23) = 12.753; *p* < 0.001) material. Results of the post hoc analysis are gathered in Table 1. The automated measurement was not part of the statistical analysis and only serves as a reference measurement.

**Table 1:** Post hoc analysis of Effectiveness measured by mean absolute error in slave coordinates for the different materials: M ≙ manual, C ≙ constraint, T ≙ trajectory

Guided milling—effectiveness											
Depth						Main visual plane					
Soft			Hard			Soft			Hard		
M1	M2	*p*	M1	M2	*p*	M1	M2	*p*	M1	M2	*p*
M	C	0.015	M	C	0.029	M	C	< 0.001	M	C	< 0.001
M	T	0.031	M	T	0.018	M	T	< 0.001	M	T	< 0.001
C	T	0.991	C	T	0.980	C	T	0.926	C	T	0.990

**Fig. 5:**
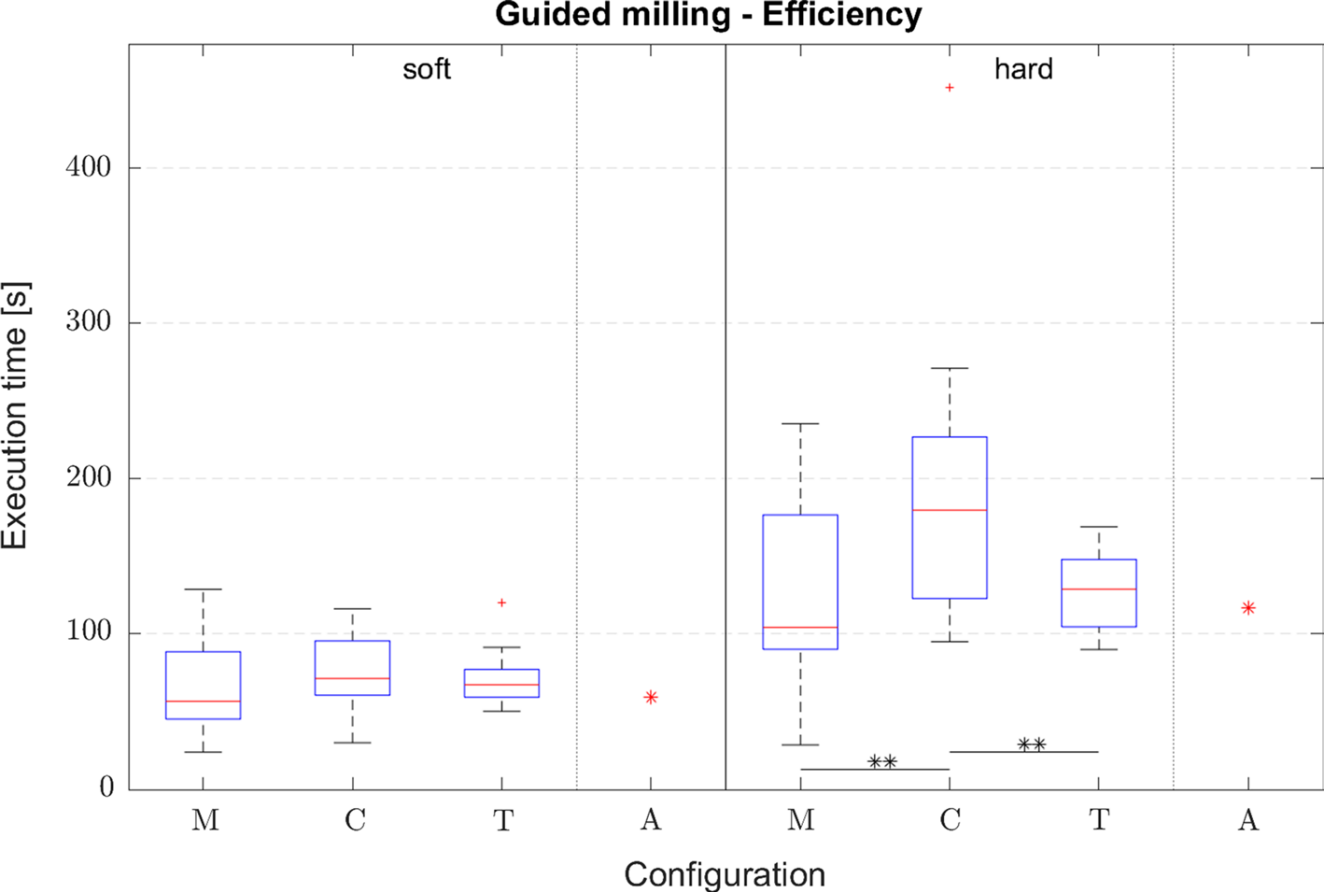
Efficiency measured by execution time for the different materials (*p < 0.05, **p < 0.01, ***p < 0.001): M ≙ manual; C ≙ constraint; T ≙ trajectory; A ≙ automated

**Fig. 6:**
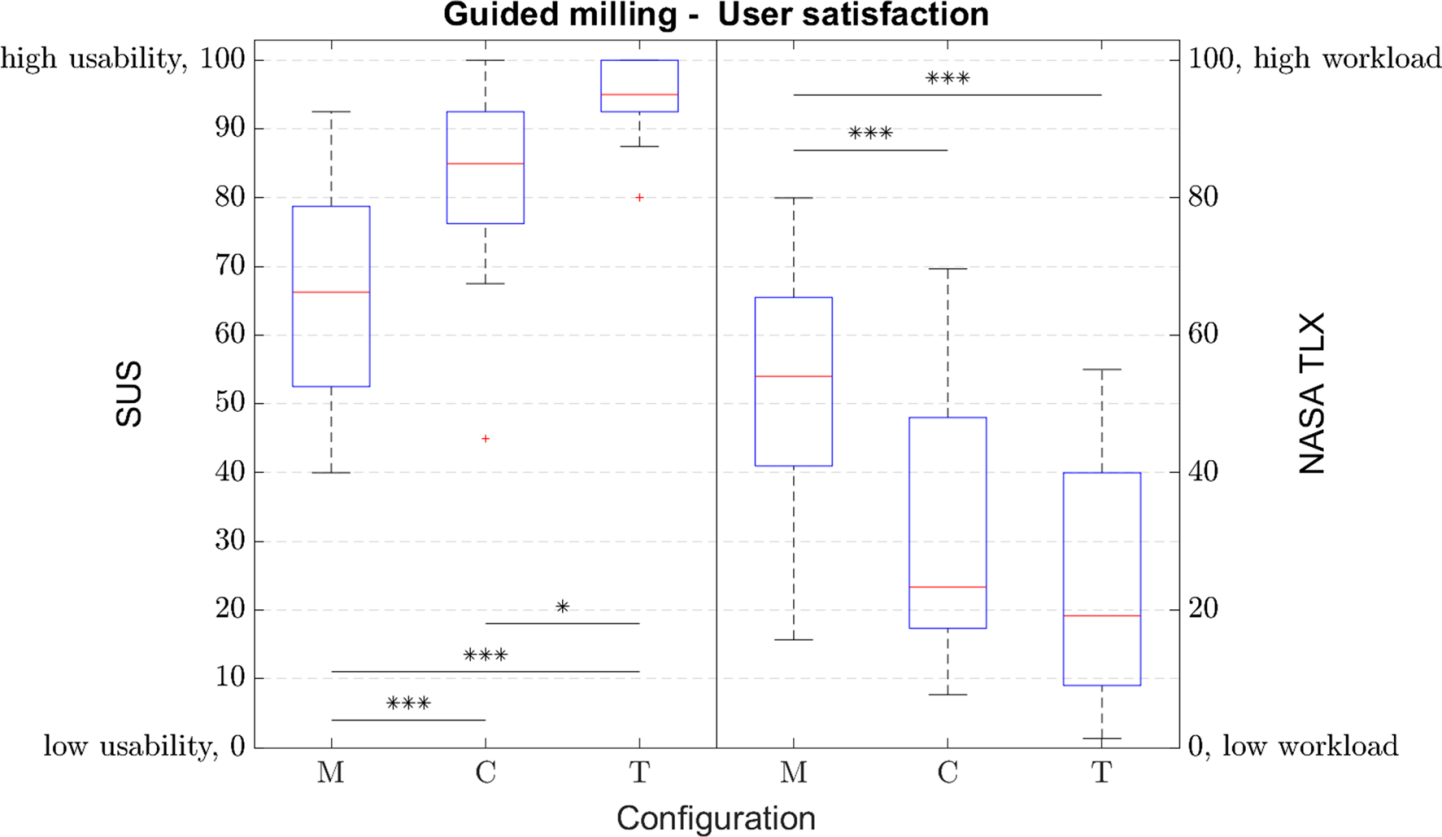
User satisfaction measured by NASA-TLX and SUS questionnaires (*p < 0.05, **p < 0.01, ***p < 0.001): M ≙ manual; C ≙ constraint; T ≙ trajectory

Figure 5 illustrates results with respect to efficiency. For soft material no significant difference is found (*F*(2,57) = 0.882; *p* = 0.420), however, a difference is found for hard material (*F*(2,57) = 5.993; *p* = 0.004). Post hoc analysis results are gathered in Table 2. Results of the user satisfaction measures can be seen in Fig. 6. A statistically significant difference is found for the results of the SUS (*F*(2,57) = 24.306; *p* < 0.001) and NASA-TLX (*F*(2,57) = 13.763; *p* < 0.001). Results of the post hoc analysis are gathered in Table 3. The statement with respect to perceived control was rated with an average rating of 5.1, 5.9 and 5.1 for the manual, constraint and trajectory modes, respectively. Furthermore, for the constraint mode participants slightly agreed that they payed close attention to the visual display (mean 4.4) and to the slave robot (mean 5.0). However, for the trajectory mode they only agreed that they payed attention to the visual display (mean 5.1), while they did not agree to be attentive to the slave motion (mean 3.5).

**Table 2:** Post hoc analysis of efficiency measured by execution time for the different materials: M ≙ manual, C ≙ constraint, T ≙ trajectory

Guided milling—efficiency					
Soft			Hard		
M1	M2	*p*	M1	M2	*p*
M	C	0.401	M	C	0.010
M	T	0.908	M	T	0.999
C	T	0.654	C	T	0.010

**Table 3:** Post hoc analysis of user satisfaction measured by NASA-TLX and SUS questionnaires: M ≙ manual, C ≙ constraint, T ≙ trajectory

Guided milling – user satisfaction					
SUS			NASA TLX		
M1	M2	*p*	M1	M2	*p*
M	C	< 0.001	M	C	< 0.001
M	T	< 0.001	M	T	< 0.001
C	T	0.023	C	T	0.463

### Depth sensitive milling

**Fig. 7:**
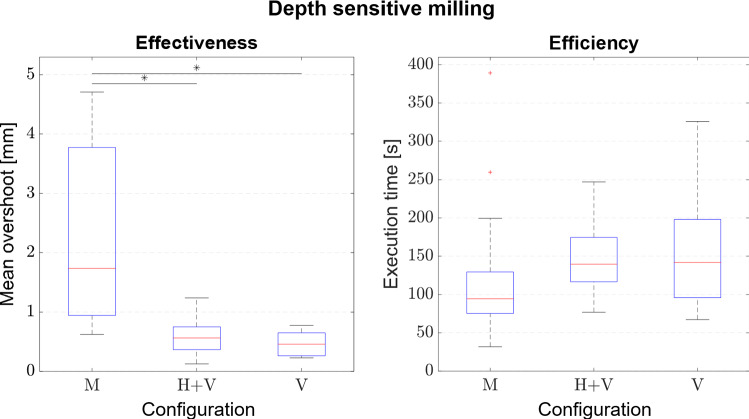
Effectiveness measured by overshoot in slave coordinates and efficiency measured by execution time (*p < 0.05, **p < 0.01, ***p < 0.001): M ≙ manual; H ≙ haptic feedback; V ≙ visually substituted force feedback

Results with respect to effectiveness are visualized in Fig. 7 (left). Due to insufficient tracking data (e.g. tracker concealment) several subjects had to be excluded resulting in 5–8 data sets per configuration for the effectiveness measure. A statistically significant difference is observed with respect to the mean overshoot (*F*(2,18) = 5.620; *p* = 0.0127). No significant difference is observed for the efficiency measure (*F*(2,57) = 1.490; *p* = 0.234) visualized in Fig. 7 (right). Results of the post hoc analysis are gathered in Table 4.

**Table 4:** Post hoc analysis of effectiveness measured by overshoot in slave coordinates and efficiency measured by execution time: M ≙ manual, H ≙ haptic feedback, V ≙ visually substituted force feedback

Depth sensitive milling					
Effectiveness			Efficiency		
M1	M2	*p*	M1	M2	*p*
M	H + V	0.042	M	H + V	0.355
M	V	0.016	M	V	0.261
H + V	V	0.788	H + V	V	0.979

**Fig. 8:**
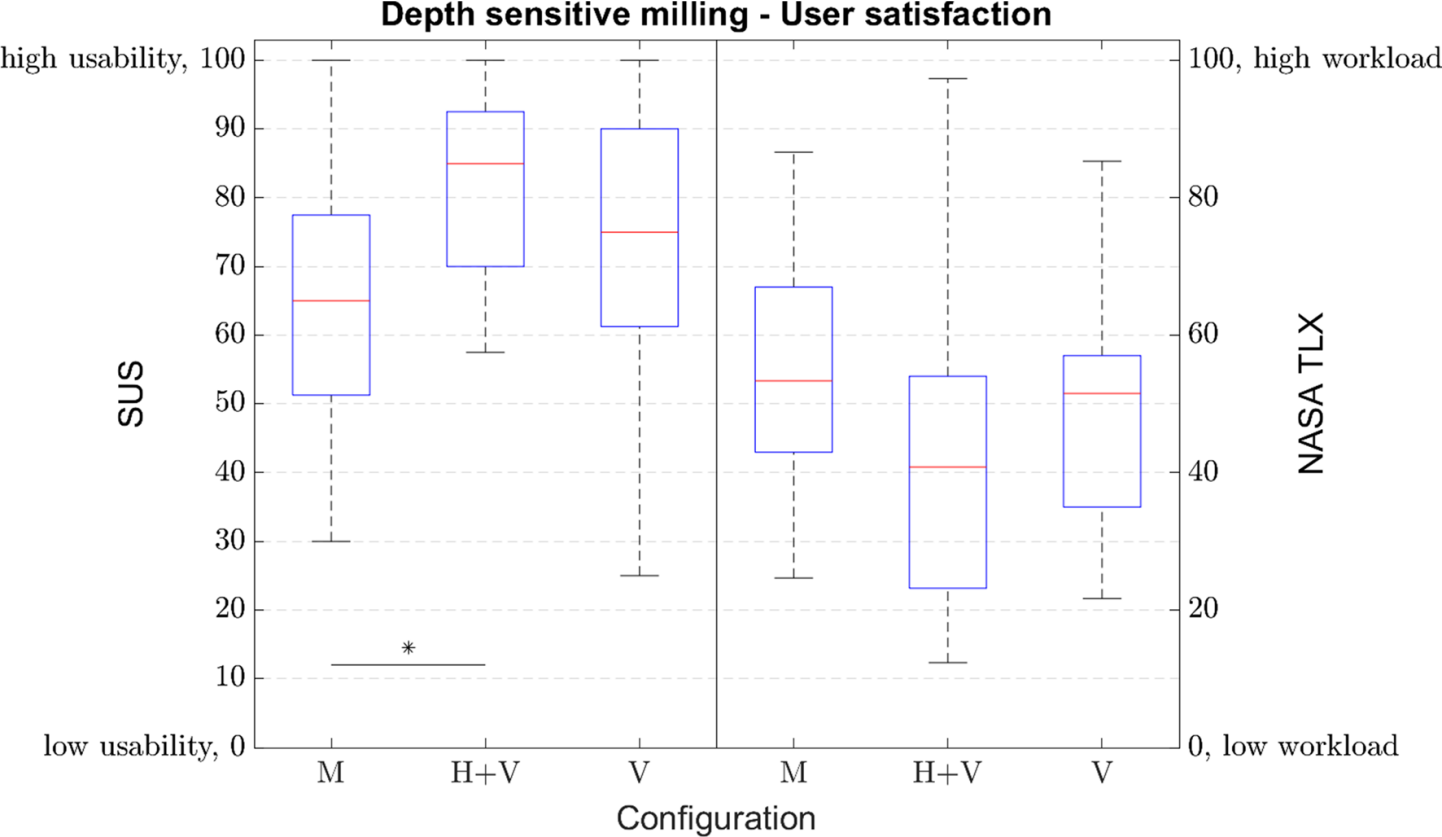
User satisfaction measured by NASA-TLX and SUS questionnaires (*p < 0.05, **p < 0.01, ***p < 0.001): M ≙ manual; H ≙ haptic feedback; V ≙ visually substituted force feedback

Figure 8 illustrates results with respect to the user satisfaction measures. A statistically significant difference could be found for the answers of the SUS questionnaire (*F*(2,57) = 3.867; *p* = 0.027), but for the NASA-TLX (*F*(2,57) = 2.579; *p* = 0.085) results no statistical difference can be proven. Results of the post hoc analysis are gathered in Table 5.

**Table 5:** Post hoc analysis of user satisfaction measured by NASA-TLX and SUS questionnaires: M ≙ manual, H ≙ haptic feedback, V ≙ visually substituted force feedback

Depth sensitive milling – user satisfaction					
SUS			NASA TLX		
M1	M2	*p*	M1	M2	*p*
M	H + V	0.020	M	H + V	0.068
M	V	0.487	M	V	0.542
H + V	V	0.247	H + V	V	0.453

In the final statements, participants supported with an average rating of 6.0 out of 7 that they could imagine milling for a prolonged time (15–30 min) with the teleoperator, while not supporting the statement with an average of 2.9 for the manual process. They did not agree (mean 2.5) that teleoperated milling was physically demanding or uncomfortable, while they agreed (mean 5.3) for manual milling.

## Discussion and conclusion

The purpose of this study was to investigate a cooperative surgical telemanipulator with respect to abstracted surgical tasks from orthopedics and neurosurgery. Three experiments were conducted to address bone tissue differentiation, guided milling and depth sensitive milling.

With the setup all participants were able to distinguish the two bone phantom materials which representing compact and cancellous bone. However, it still seems to be slightly easier in direct manual than in telemanipulated interaction. One option to improve the differentiability of materials with the telemanipulation setup would be to incorporate force or stiffness scaling since signals can be modulated before they are mirrored back to the user on the master device. This way differences may become more apparent. Regarding the perceptive similarity between the manual and the teleoperated feedback participants slightly agreed that materials felt similar with the same score for hard and soft material. Hence, perceived differences are unlikely to be due to the different control architectures. However, it potentially can be attributed to the reduced dimensionality of the force feedback, which, however, according to [42] should not impede performance, as well as low-pass filtering of the sensor signal to reduce vibrations and sensor noise. Even though the latter may lead to perceived differences this can also be positive in case vibrations are unwanted and hinder controllability of the burr, which will be further discussed for the following experiments.

For the guided milling experiment, reduced errors are found for both telemanipulated configurations, which confirms findings of previous studies [11, 33] and is in line with results of [43]. Comparing the result to a study on manual milling of neurosurgeons (mean depth and lateral error of 0.7 mm [34]) shows that novice users can achieve a similar or better accuracy when using the teleoperation setup. Evaluating the efficiency of the different configurations shows no differences in execution time for the soft material. However, it should be noted that in the trajectory mode milling was always performed in two layers, whereas the strategy of participants using the constraint mode was mostly to work in one layer. For hard material, where subjects had to work in multiple layers a decrease in execution time for the constraint mode becomes apparent. Comparing the trajectory mode with the exemplary automated execution shows that results for effectiveness as well as efficiency are in close proximity to the automated execution indicating that slave side accuracy as well as the chosen machining parameters (i.e. velocity, depth of cut) greatly influence results. Therefore, if efficiency needs to be increased higher velocities can be chosen, as for example feed rates for hard material of up to 5 mm/s are reported in the literature [36], or the depth of cut for the soft material could be increased to 3 mm, which would reduce the execution time by about half. Results of the perceived system usability and the perceived workload show an improvement for both teleoperated modes. The perceived system usability shows an additional improvement for the trajectory mode compared to the constraint mode. However, in the constraint mode participants felt a little more in control compared to the manual or the trajectory mode. That less intrusive controllers result in more perceived control was also found in [44], however, only the option to switch off assistances can increase the perceived influence [33]. The comparably low perceived influence on the manual execution was justified by participants by the statement that they did not feel in full control over the tool due to the acting milling forces and vibrations. Interestingly, the assistance mode also influenced the focus of attention, and while participants focused predominantly on the robot and situs for the constraint guidance, they indicated to be more focused on the GUI in the trajectory mode. This could be due to the fact that the only indication of the course of the trajectory was on the GUI. To shift focus more towards the robot a visual feedback closer to the robot, for example by means of augmented reality display, could be considered.

Results of the depth sensitive milling task show a reduction in the mean overshoot for both telemanipulated configurations. However, no difference is observed between visually substituted force feedback and additional haptic feedback. Effects of haptic feedback in the literature are controversial and while [45] finds improvements with respect to effectiveness, [46] does not. The negative effect which was observed in [33] due to a rapid decline in the force signal is less apparent in this investigation. According to [47] the advantage of telemanipulation systems with respect to effectiveness can be mainly attributed to motion scaling, which is not possible with any other cooperative robotic system [3]. With respect to efficiency, no statistically significant differences can be found; however, tendencies indicate manual execution being slightly faster than both teleoperated modes, which is in line with results of [45, 47], who found significantly faster manual execution times compared to telemanipulation. The addition of haptic feedback showed no difference. In the literature the effect of additional haptic feedback is again controversial with respect to efficiency and while [45] finds no difference, [48] finds a decrease in execution time if force feedback is added. Scaling between master and slave cannot be made accountable for the increased time using a telemanipulation system according to [47]. Therefore, to improve efficiency of the setup the velocity during the first two layers, which are outside the security offset can be guided as in the experiment on guided milling and if necessary the milling velocity can be further increased to reach similar execution times as manually. This should also not impair the effectiveness measure, since the execution of the last layer within the security offset determines the mean overshoot. Even though improvements due to haptic feedback from the slave with respect to effectiveness and efficiency remain controversial, improvements with respect to a reduction in the perceived workload or an increase in the perceived system usability can be seen, though significance is only reached for the SUS score. Therefore, the addition of haptic feedback seems to lead to a subjective improvement, which is in accordance with [33], however, no performance enhancements can be found.

Limitations of the study include that some data of the effectiveness variables had to be excluded for the guided milling and the depth sensitive milling experiment. Participants knew the respective assistance mode that was currently active in each test mode, which could have influenced the subjective evaluations, and that participants were not surgeons. Furthermore, since assumptions of ANOVA could only be checked visually due to the limited number of participants statistical results have to be treated with some reservation. Perceived criticality of the task is hard to simulate in an experimental lab setting but of course will have an impact on the efficiency of an unguided manual milling task. Learning effects could have influenced results, however, due to randomization of trials within each experiment this constitutes a random rather than a systematic error. Nevertheless, as experiments were always conducted in consecutive order, learning effects could have influenced results of the second and third experiment. Since participants had a high technical affinity subjective evaluations could be skewed towards the more technically advanced modes.

All things considered, results suggest that a cooperative surgical telemanipulator for orthopedics and neurosurgery can improve effectiveness close to an automated execution and improve user satisfaction while not degrading efficiency. Haptic feedback can be used to differentiate compact and cancellous bone, however, performance improvements remain controversial and only subjective benefits are obeserved. Therefore, system designers should thoroughly evaluate whether the subjective benefits outweigh the higher system complexity. Additionally, the increased amount of hardware needed for a cooperative telemanipulator compared to other cooperative robots (i.e. hands-on or handheld devices) should be considered when designing a system for a particular application [3, 29] Overall participants preferred using the telemanipulator since it is less physically demanding/uncomfortable compared to the manual process and their performance is simultaneously improved. In addition, the surgeon is part of the control loop and is able to adjust the surgical plan based on his/her expertise at any time.
